# Surfactant Semiconductors as Trojan Horses in Cell‐Membranes for On‐Demand and Spatial Regulation of Oxidative Stress

**DOI:** 10.1002/adhm.202202290

**Published:** 2023-01-13

**Authors:** Marian Jaschke, Masina Plenge, Marius Kunkel, Tina Lehrich, Julia Schmidt, Kilian Stöckemann, Dag Heinemann, Stephan Siroky, Anaclet Ngezahayo, Sebastian Polarz

**Affiliations:** ^1^ Institute for Inorganic Chemistry Leibniz University Hannover Callinstrasse 9 30167 Hannover Germany; ^2^ Institute for Cell Biology and Biophysics Leibniz University Hannover Herrenhäuser Str. 2 30419 Hannover Germany; ^3^ Department of Chemistry University of Konstanz Universitaetsstrasse 10 78457 Konstanz Germany; ^4^ Institute of Horticultural Production Systems and the Cluster of Excellence PhoenixD Leibniz University Hannover Welfengarten 1 30167 Hannover Germany

**Keywords:** biocompatible amphiphiles, reactive oxygen species, self‐assembly, semiconductor surfactants

## Abstract

Oxidative stress is a cause for numerous diseases and aging processes. Thus, researchers are keen to tune the level of intracellular stress and to learn from that. An unusual approach is presented here. The methodology involves multifunctional surfactants. Although their molecular design is nonbiological—a fullerenol head group attached covalently to pi‐conjugated dyes—the surfactants possess superior biocompatibility. Using an intrinsic fluorescence signal as a probe, it is shown that the amphiphiles become incorporated into the Caco‐2 cells. There, they are able to exhibit additional functions. The compound reduces cellular stress in dark reaction pathways. The antagonistic property is activated under irradiation, the photocatalytic production of reactive oxygen species (ROS), resulting in cell damage. The feature is activated even by near‐infrared light (NIR‐light) via a two‐photon process. The properties as molecular semiconductors lead to a trojan horse situation and allows the programming of the spatial distribution of cytotoxicity.

## Graphical abstract

Surfactants with a fullerenol head and dye tails are biocompatible and can be detected and located in cells by fluorescence probe properties. Two functions are opposed, the protection of cells in the dark versus photocatalytic generation of ROS species. The interplay allows the creation of spatially resolved stress patterns.

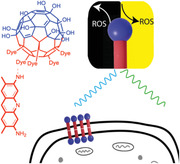

## 1 Introduction

Surfactants deviating significantly from biological design principles^[^ ^1^ ^]^ often are harmful to cells as they destroy the cellular membranes.^[^ ^2^ ^]^ However, depending on their chemical nature and the so‐called packing parameter describing the molecular geometry, surfactants are capable of forming double‐layer vesicles, respectively liposomes, which resemble the membranes of cells. Such surfactants with lipid‐like behavior have successfully been applied in drug‐delivery, and the so‐called niosomes (nonionic surfactant based vesicles) have proven to be particularly valuable.^[^ ^3^ ^]^ Self‐assembled capsules have become important as carriers for mRNA in vaccines.^[^ ^4^ ^]^ Those studies show that suitable vesicles can be taken up by cells, where they may accumulate and eventually be metabolized. The surfactant was in the mentioned cases the amphiphilic entity forming the capsule by self‐assembly but not more.

Recent research has led to the emergence of more functional surfactants with properties going beyond amphiphilicity.^[^ ^5^ ^]^ Two particular important classes are catalytically active surfactants and amphiphilic compounds with semiconductor properties leading to photochemical activity.^[^ ^6^ ^]^ It is proposed in literature that such functional surfactants, if they were biocompatible, could be applied for a more complex nanomedical treatment or even for theranostics.^[^ ^7^ ^]^ We envision that, in addition, photoactive semiconductor surfactants incorporated by cells can be used for advanced fundamental biological studies on cellular stress and ageing. The external control of the reactive oxygen species (ROS) concentration inside living organisms is key to achieve a deeper understanding of ageing processes, but to actually realize such a control remains a major challenge.

At moderate concentrations ROS like superoxide, hydroxyl radicals and others play an important role in various cellular redox and signaling processes.^[^ ^8^ ^]^ However, an often‐occurring source of various diseases is an increase in oxidative stress on a cellular level caused by ROS due to an insufficient quenching metabolism. The natural protection mechanism using scavenging enzymes like manganese superoxide dismutase no longer copes with the elevated ROS concentration leading to apoptosis of nearby tissue like neurons and other cells.^[^ ^9^ ^]^ A rise in cellular ROS concentration leads to DNA and structural damage in cells. Consequently, oxidative stress is a major factor in neurodegenerative disorders, diabetes, cardiovascular disorders, cancer, and other chronic and age‐related diseases^[^ ^10^ ^]^ The in vivo generation of high ROS concentration^[^ ^11^ ^]^ and a resulting cell apoptosis is wanted for the photodynamic therapy of cancer^[^ ^12^ ^]^ or against microbial infections.^[^ ^13^ ^]^

**Scheme 1:**
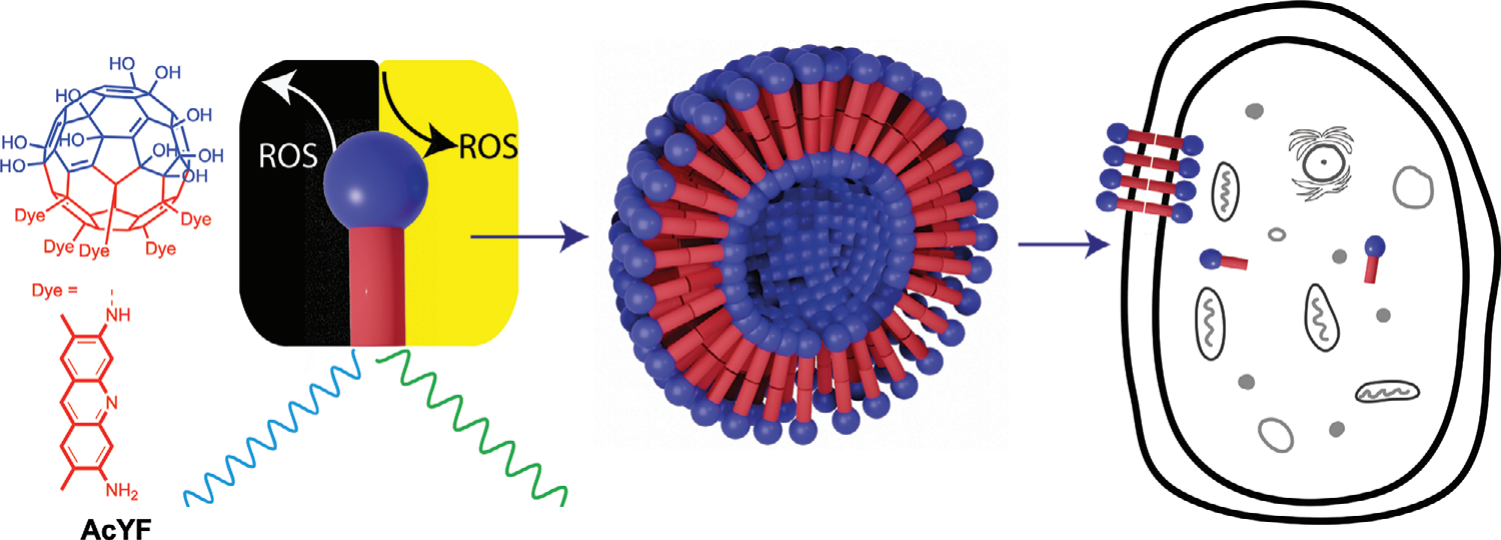
Molecular structure (left) of the semiconductor surfactant AcYF with a fullerenol head group (blue) and 5 x acridine yellow side chains attached to it (red). In addition to self‐organization into vesicles (middle), the surfactant fulfills several additional functionalities at once. It is biocompatible and it is a fluorescence probe which allows to prove its integration and accumulation into living cells (right) and positional fixing. Under irradiation it produces ROS species and consumes ROS in the dark.

The approach described in the current paper involves smuggling a compound into living cells, which enables an external control of the ROS level. Our idea is that a multifunctional semiconductor surfactant can fulfill all required tasks and more (see **Scheme** **1**).

## 2 Results and Discussion

The basis for the work presented herein is our previous result on a novel class of surfactants containing a fullerenol head group.^[^ ^14^ ^]^ This (hydrophilic) head is C_60_ which is hydroxylated only on one side in a Janus‐type fashion with ≈20 x OH groups. Precisely five organic residues can be attached to the other hemisphere of the fullerene as the hydrophobic entity.^[^ ^14b^ ^]^ After the establishment of compounds with alkyl side‐chains,^[^ ^14a^ ^]^ we have already shown that fullerenol‐dyad derivatives act as semiconductor surfactants.^[^ ^14c^ ^]^ However, the semiconductor surfactants have never been used in a biological context. Therefore, we will first investigate the multifunctionality visualized in Scheme 1, check for the biocompatibility next, probe the protection of cells by the surfactant (→ ROS deactivation), and finally we will study the triggered and spatially controlled increase of the oxidative stress level (→ ROS production).

### 2.1 Multifunctionality of the Semiconductor Fullerenol‐Dyad Surfactants

**Figure 1:**
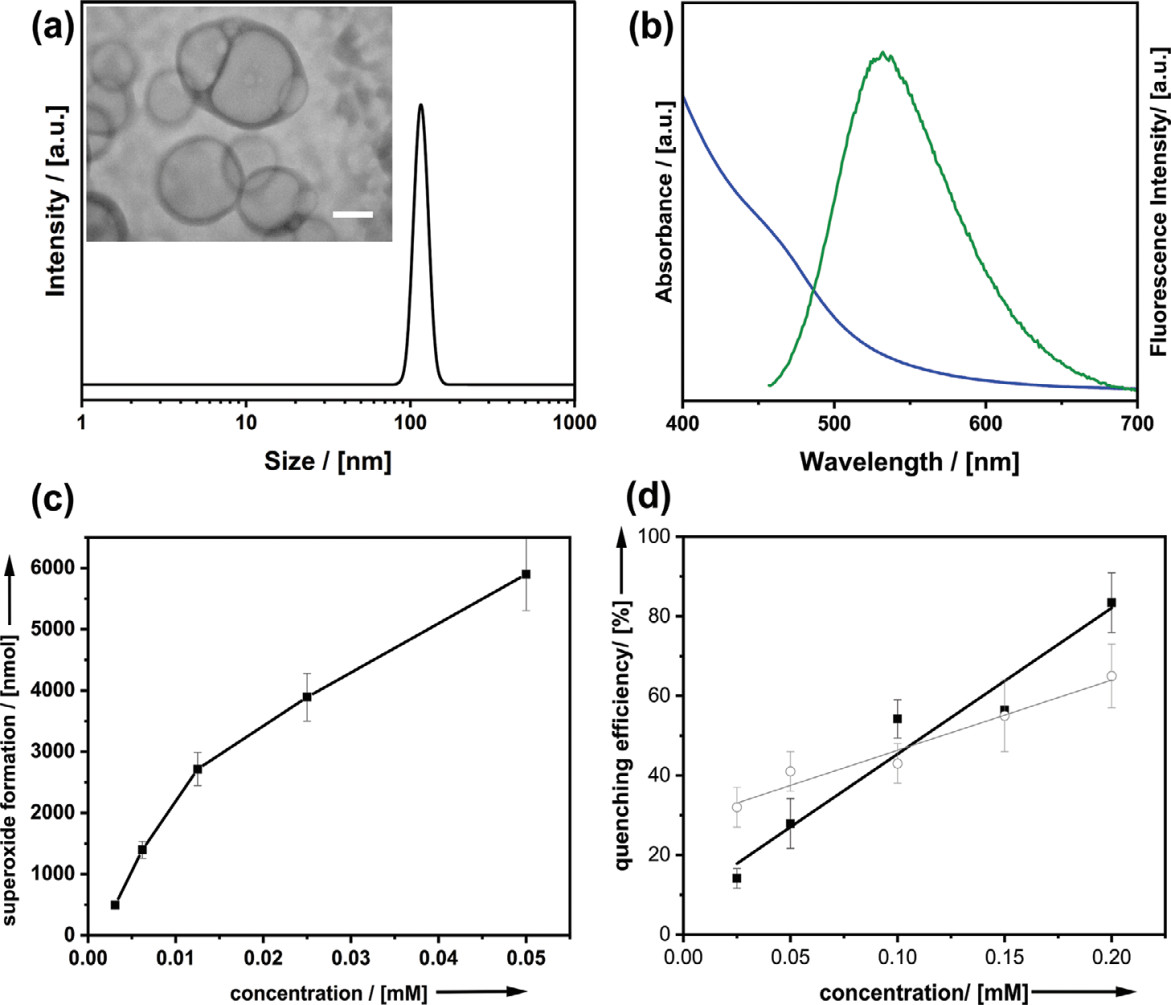
a) DLS and cryoTEM (inset; scalebar = 100 nm) of AcYF in water at c = 0.4 mm. b) UV–vis absorption spectrum (blue) and fluorescence spectrum (green) of AcYF. c) superoxide formation determined by NBT‐assay as a function of the concentration of AcYF. d) deactivation of ROS‐species by AcYF (squares) in the dark and quercetin (circles) as a reference.

The molecular compound (AcYF) containing acridine‐yellow residues attached covalently to the fullerenol head group is at the center of the current publication as it features a set of five different functionalities: amphiphilicity and capability to form self‐assembled aggregates a), fluorescence b), photocatalytic ROS production activity c), nonradiative ROS deactivation activity d) and biocompatibility e). **Figure** **1** summarizes selected analytical data to demonstrate the functionalities (a–d). The prime property of AcYF is amphiphilicity, meaning it spontaneously forms self‐organized aggregates above a critical concentration (cc). The water–air interface is saturated at *c* ≈ 0.2 mm = cc according to concentration‐dependent surface tension measurements (see Figure S1, Supporting Information). The aggregates found at slightly higher concentration (*c* = 0.4 mm) are investigated by dynamic light scattering (DLS) and transmission electron microscopy (TEM) under cryogenic conditions (Figure 1a). Considering that the molecular extension of the surfactant is only ≈2 nm, the objects with an average diameter of ≈110 nm are too large for micelles (≈ double of the surfactant length) but are consistent with vesicles. The latter conclusion is consistent with the low contrast of the spherical objects seen in cryo‐TEM rather than with the formation of compact objects.

In comparison to the optical absorption spectrum of acridine yellow (band at *λ*_abs,max_ = 442 nm), AcYF is characterized by a semiconductor‐like absorption edge starting at *λ*_abs_ ≈ 500 nm (Figure 1b, blue). There is a broad emission band with *λ*_em,max_ = 530 nm found by fluorescence spectroscopy. Therefore, one can utilize the compound as a fluorescent dye. However, with a quantum yield of 0.60% only a fraction of the absorbed light goes into fluorescence emission. Because the majority of the energy should be channeled into photochemical processed, maximization of the fluorescence quantum yield was not intended and actually is also not wanted. Therefore, one cannot compare AcYF with fluorescence probes which have been developed for this purpose alone. The major portion of the energy is canalized into the formation of the superoxide radical (^3^O_2_ + e_ex_^−^ → ^3^O_2_^•−^) which was probed as described by literature procedures^[^ ^15^ ^]^ using a nitrotetrazolium blue essay (NBT). It can be seen (Figure 1c) that AcYF is highly active in superoxide generation even at very low concentration (<< cc).

The formation of singlet oxygen under irradiation (^3^O_2_ → ^1^O_2_) is another possible channel of photocatalytic activity, which is well documented for dyes like acridine yellow. The activity in ^1^O_2_ production can be tested by investigation of the bleaching of p‐nitrosodimethylaniline (pNDA) at 440 nm using imidazole as a selective acceptor to singlet oxygen.^[^ ^16^ ^]^ Our results shown in Figure S2 (Supporting Information) indicate that AcYF is unable to produce ^1^O_2_. This inability can be seen as an advantage because ^1^O_2_ would attack the *π*‐systems of the dye, which essentially would cause self‐destruction. In accordance to these arguments, AcYF is stable against photobleaching as shown in Figure S3 (Supporting Information).

It is interesting to note that fullerenols behave oppositely in the dark,^[^ ^14^, ^17^ ^]^ meaning they catalyze the elimination of an entire range of ROS species such as O_2_^•−^, OH^•^, or H_2_O_2_. The activity of AcYF in ROS deactivation was also probed by an NBT assay and is shown in Figure 1d. As expected, there is a linear correlation of ROS quenching efficiency with the concentration of the surfactant. At *c* > 0.2 mm (= cc) quenching is almost quantitative and it outperforms the commercial gold‐standard quercetin.

### 2.2 Biocompatibility and Positioning Semiconductor Fullerenol‐Dyad Surfactants in Living Cells

Because of the low cytoxicity of fullerenol‐alkyl surfactants,^[^ ^14a^ ^]^ we hoped that AcYF is highly compatible with living cells as well. The human cell line Caco‐2 was used as a test system. Caco‐2 cells are a model of the intestinal epithelium which is known to be particularly prone to oxidative stress.^[^ ^18^ ^]^

**Figure 2:**
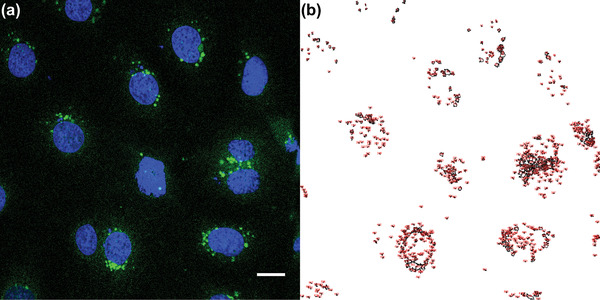
a) Confocal laser scanning microscopy image of Caco‐2 cells loaded with the surfactant AcYF at *c* = 10 µm for *t* = 1 h and stained with Hoechst 33 258 (blue, nucleus) scalebar = 20 µm. b) The green fluorescence indicates vesicle‐like structure containing AcYF. Of note the micrograph were taken after the removal of AcYF containing solution. The data show AcYF molecules accumulated in the cells.

In first step, it is important to demonstrate if and how AcYF interferes with Caco‐2. Now, the optical characteristics of AcYF come handy. It is spatial distribution (→green fluorescence) can be clarified using confocal laser scanning fluorescent microscopy (CLSM). When the nucleus is stained with Hoechst 33 258 (→blue fluorescence), one can obtain deeper insights about the location of the surfactant in vesicle‐like structures in the cells (green dots). For the loading of the cells with the surfactant, an aqueous solution containing a defined concentration of AcYF *c* = 1 × 10^−9^–2.5 × 10^−7^ mol L^−1^, was given to the cell culture medium with cells adherent on glass cover slips and maintained in culture medium (37 °C) for different times *t* = 0.25, 1, 3, 6 h. Finally, CSFM micrographs (**Figure** **2**) were taken after the replacement of AcYF containing culture medium by fresh AcYF‐free culture medium.

One can see that the distribution of AcYF is not homogenous as it would be for pure, dissolved surfactant. AcYF molecules are assembled in the cells in vesicle‐like structures (Figure 2; and Figure S5, Supporting Information). A greenish background can be seen suggesting presence of AcYF molecules in the cell membrane. One can conclude that the cells show a pronounced tendency for internalization of the surfactant in vesicle‐like structures. After longer times (3, 6 h) the fraction of AcYF in the vesicle‐like structure in the cytoplasm increased. This effect can be explained as follows: cells continuously recycle the membrane by an endocytotic activity.^[^ ^19^ ^]^ Endocytosis is a normal cellular process by which cells internalized materials liquid or large particles, e.g., bacteria or large vesicles from the external milieu. The process starts with self‐folding of the cell membrane to form a pit around the material to be engulfed. After closing the borders of the pit trapping in thereby the material, a vesicle pinches off in the cytoplasmic space. The vesicle is then directed to the lysosome, where the engulfed material and the vesicular membrane are degraded and eventually recycled. The endocytosis participates thereby also in membrane recycling. In our case the finding of the surfactant molecule into the cytoplasmic space (Figure 2) in vesicle‐like structures suggests that the surfactant may have been associated with the cell membrane and internalized afterward by endocytic activity. It is also possible that the surfactant molecules were internalized as preformed vesicles. Either way, Figure 2 shows in cells accumulated molecules of the AcYF surfactants en‐route to the lysosomes.

**Figure 3:**
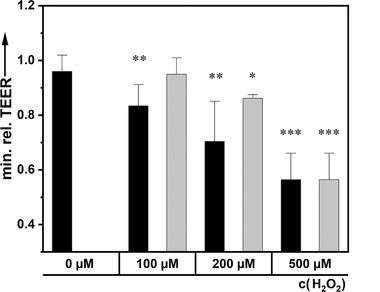
AcYF induced a protection of the cellular barrier function against oxidative stress. The figure shows the relative reduction of the cell monolayer TEER induced by H_2_O_2_ in absence (control, black) and in presence of 100 µm fullerenol variants (AcYF, gray). The experimental results were normalized to results obtained from cells cultivated in parallel without H_2_O_2_ and AcYF. The data are given as average ± s.d for 5 independent cultures. One‐way ANOVA analysis was applied for statistical significance to control cells (*: *p* < 0.05¸**: *p* < 0.01¸***: *p* < 0.001).

A further advantage of Caco‐2 cells is their capacity to form a monolayer with a good barrier with high transepithelial electrical resistance (TEER>1000 Ω cm^2^), when cultivated in inserts of transwell system. This offers an excellent possibility to access the effect of compounds on the viability of the cells by following an essential function of the epithelial cells. In this context, we observed that presence of AcYF for 6 h to load the cells with the surfactant did not affect the TEER of the cell monolayers (**Figure** **3**). Together with the observation that cells loaded with AcYF maintained their morphology, their mobility (Figure **2**; given as movies in the Supporting Information) and their proliferative activity (Figure S5, Supporting Information), we can affirm that for the loading time of 6 h the cells are highly tolerant and stable against AcYF despite its pronounced nonbiological nature.

Moreover, the AcYF molecules accumulated in the cells during the loading time do not affect essential cellular functions. Even if we show that the cells continued to proliferate after a loading time of 96 h (Figure S5, Supporting Information), we cannot with the present report answer to whether the surfactant could be maintained in the cell culture media, nor to how AcYF is metabolized and may affect cellular function like gene expression in long term. To answer to these questions will involve detailed considerations far beyond the scope of the present paper. The present report shows that a loading time of 6 h was enough to allow an accumulation of the surfactant without affecting the physiology of the cells. In the following, we analyze whether in the cells accumulated surfactant molecules could be used to protect against oxidative stress or to generate oxidative sepsis in the cells.

### 2.3 Surfactant‐Aided Protection Against Oxidative Stress

Next, it will be important to investigate if the activity of AcYF regarding ROS species is maintained also when it has been incorporated into the cells. We concentrate on a potential support of the epithelial barrier function against oxidative stress by the incorporated semiconductor surfactants. H_2_O_2_ is a representative of oxidative signals in tissue with a plethora of cellular functions ranging from oxidation of proteins, induction of gene expression and cell as well as tissue damaging.^[^ ^20^ ^]^ In epithelial cells H_2_O_2_ may attack the barrier function which can easily be followed by measuring TEER of the monolayers.^[^ ^21^ ^]^ In tissue H_2_O_2_ as representative of oxidative reaction is mostly maintained at low systemic concentrations that vary between 2.5 and 25 µm.^[^ ^22^ ^]^ This is due to a low production and mostly to a high efficiency activity of the different cellular degradation mechanism that achieved a containment of the produced H_2_O_2_ in the vicinity of production and for a reduced time avoiding thereby deleterious effects of the oxidative molecules. Relatively high concentrations in range of hundreds of micromolar are produced in response to massive stimulation of the immunological system or to a long lasting hypoxia.^[^ ^22^ ^]^ In these cases the oxidative signals are considered as oxidative stress that may affect the function of tissue as shown in this report in which we show that around 100 µm H_2_O_2_ may affect the epithelial barrier function. The effect of different H_2_O_2_ concentrations on the barrier function of Caco‐2 cells cultivated in inserts and a potential protection effect of the semiconductor surfactant AcYF was investigated by TEER (Figure 3).

**Figure 4:**
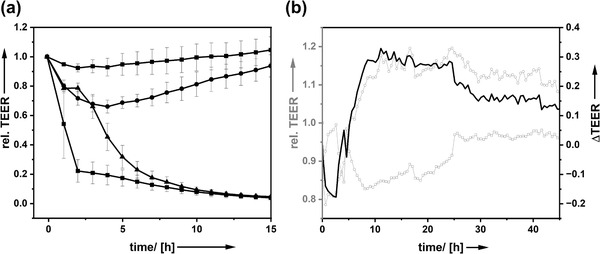
a) Time‐dependent transepithelial electrical resistance (TEER) for the application of different levels of H_2_O_2_ (c = 100 µm, squares; 500 µm, circles; 1 mm, triangles; 5 mm, hashes;) protected by the semiconductor surfactant fulleronol variants. The data we normalized to data obtained from to non‐ treated cells. b) Comparison of TEER (grey) of Caco‐2 exposed to *c* = 200 µm H_2_O_2_ in presence of surfactant (hollow spheres) and without the surfactant as a reference (hollow squares). The difference in protection against oxidative stress is also shown as ΔTEER (black line).

The cells formed a tight monolayer with very good barrier function as attested by the high TEER above 800 Ω cm^2^. AcYF was applied apically. This application correlated with a slight perturbation of the barrier, which was resolved within 1 h. After incubation of the cells with AcYF for 6 h, residual AcYF was removed and washed off the cells, following exposure to H_2_O_2._ At that time the surfactant has been incorporated into the cells. The control contains no surfactant. The time‐dependent TEER signal is plotted as a function of time in **Figure** **4**a. High H_2_O_2_ concentrations (> 1 mm) destroy the barrier function as shown by the strong and almost instantaneous reduction of the TEER by a factor of 70%, regardless if AcYF is present or not. After addition of H_2_O_2_, the TEER value decreases more and more which shows that the cells are damaged irreversibly. A lower concentration of H_2_O_2_ (≦ 500 µm), expectedly, leads to a less significant dip of the TEER. Compared to a surfactant‐free reference sample, we see that fast(er) recovery takes place for cells treated with AcYF shown in Figure 4b as the positive values for the ΔTEER indicate. One can clearly see a protective effect of AcYF incorporated in Caco‐2 (see also Figure 3) confronted to 100 µm H_2_O_2_ or even 200 µm H_2_O_2_. The experiments prove that the surfactant could be used in condition of pathological production of H_2_O_2_ to protect the tissue.

### 2.4 Surfactant‐Aided Generation of Oxidative Stress

**Figure 5:**
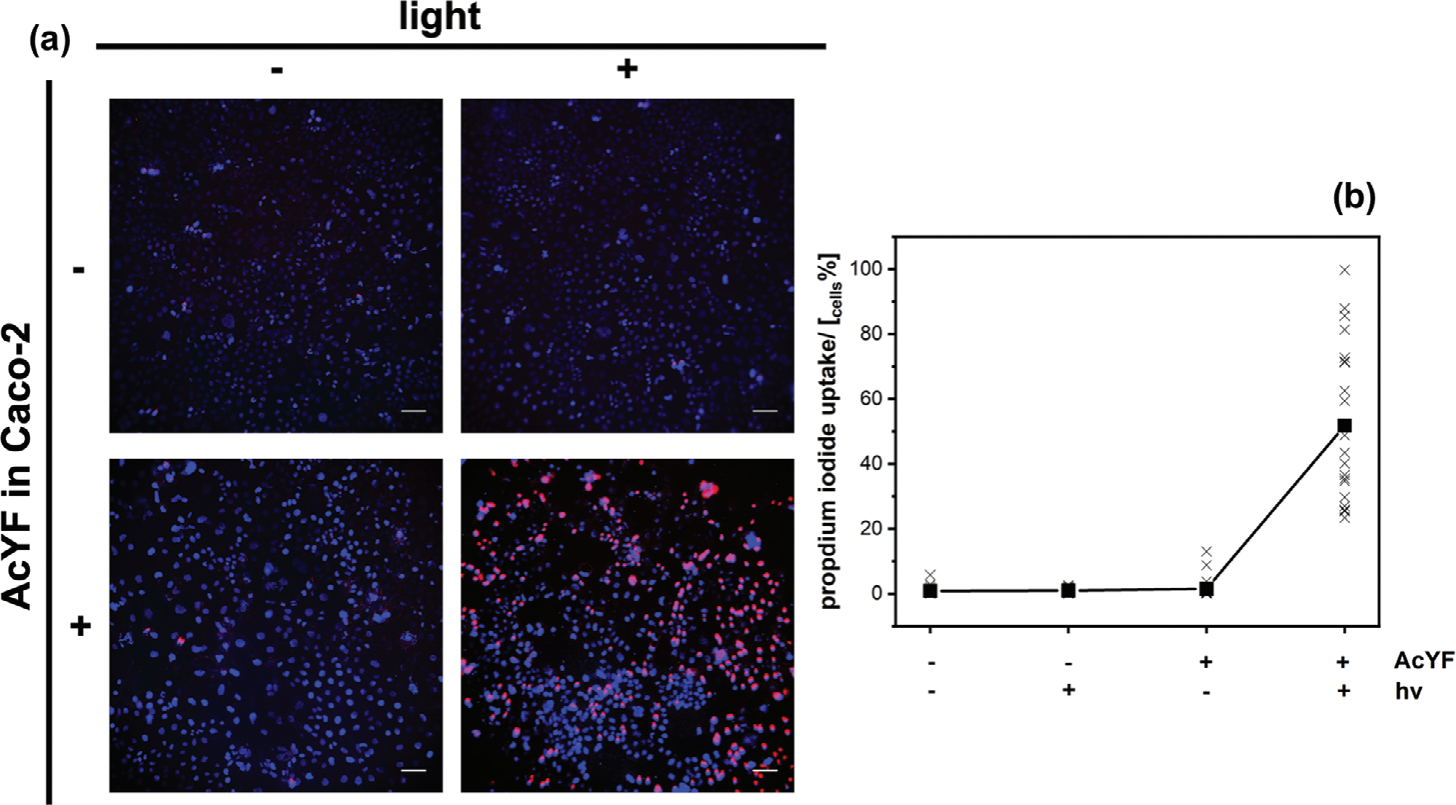
AcYFME LP measurements. The reaction of Caco‐2 cells (blue = intact marked with Hoechst 33 258; purple = damaged marked with PI) depending on the radiation with light (+ ≅ 543 nm; – ≅ dark) and the incorporation of AcYF (+/−) as determined by fluorescence microscopy images (a; scalebar = 100 µm) and their quantification b). See also control experiments given in Figure S5 (Supporting Information).

It has been shown above that another functionality of the surfactant is its activity in the photocatalytic formation of ROS species. For the investigations in cells, we use a variant of the gold nanoparticle‐mediated laser perforation (GNOME LP) technique available in our laboratory.^[^ ^23^ ^]^ Treatment of cells with laser may induce a reversible permeabilization of cells that is not pathological. This reversible permeabilization is resolved in a relatively short time. However, if the permeabilization persist over hours, it becomes a pathological process, that correlates with cell death.^[^ ^23^, ^24^ ^]^ In the modified methodology, AcYF takes over the part of the gold nanoparticles (⇒ AcYFME LP) and allow a permeabilization of the cells upon photoactivation. The setup is equipped with a laser emitting at *λ* = 532 nm. The laser radiation is absorbed by AcYF at the low energy tail of the semiconductor band‐edge (see Figure 1b). A pathological dose and duration were applied. As it is known from literature^[^ ^25^ ^]^ and our own work^[^ ^14c^ ^]^ that fullerenol compounds can produce ROS under irradiation, together with the unambiguous proof that AcYF is present inside the cell and nowhere else, ROS generation is located inside the cell as well. Quantification using a ROS assay kit was not possible at this time. However, the production of ROS was accessed by the quantification of permeabilized cells that were indicated by the capacity to take up a membrane‐impermeable dye like propidium iodide (PI). The occurrence of a purple fluorescence signal shows PI has reacted with the intracellular DNA of Caco‐2. The evaluation of the pathological permeabilized cells was performed 1 h after the laser application. Many cells were already lysed. Therefore, the evaluation underestimated the killing effect of the produced ROS. However, in‐line with the formulated hypothesis, ROS‐formation and cell damage occurs only if Caco‐2 contains AcYF and if the system is exposed to light (**Figure** **5**).

The applied visible wavelength (*λ* = 532) is well suited for 2D in vitro cultures, but would only provide limited penetration depth in biological tissue and result in strong absorption by oxygenated Hb within the blood. Accordingly, excitation with NIR‐light within the therapeutic window (650–1350 nm) would be beneficial regarding possible future in vivo applications. Therefore, we investigated the possibility for two‐photon excitation of the fluorescence and the ROS‐production pathways using a NIR femtosecond laser. It was found, that visualization was possible at low intensities with *λ* = 800 nm. At these settings, no signs of cytotoxicity were observed. Using slightly more intense illumination at *λ* = 950 nm resulted in a dose‐dependent apoptotic appearance of the irradiated cells loaded with AcYF, indicating substantial ROS production, whereas no detrimental effects were observed control cells only counterstained with Acridine orange; see Figure S6, Supporting Information.

### 2.5 Spatially Resolved Cytotoxicity Due to Borderline Character of the Surfactants

**Figure 6:**
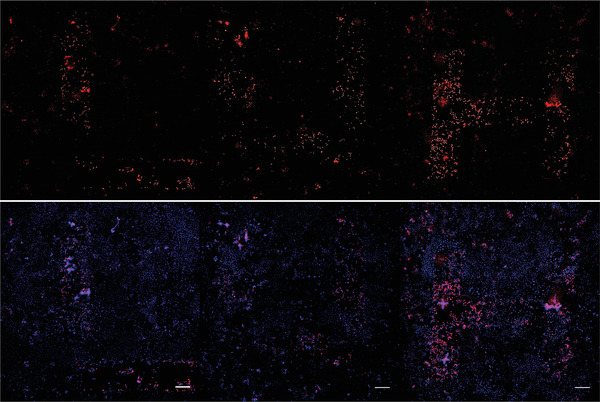
(bottom) Fluorescence microscopy analysis (scalebar = 100 m) of the local light treatment of a monolayer of Caco‐2 cells containing AcYF surfactants; blue = intact marked with Hoechst 33 258; purple = damaged marked with PI. (top) optical revision showing only dead cells in red color.

Up to this point, it has been shown that the AcYF surfactants accumulated in the cells show diametral behavior. They effectively protect cells against oxidative stress, but at the same time they are trojan horses. Light as a trigger induces the formation of ROS inside the cells, and as a consequence cell death. The interplay of the two factors together should enable to create a spatially very defined pattern of cytotoxicity. As a proof‐of‐concept we present **Figure** **6** which was created by writing the letters LUH using the described AcYFME LP setup applied on a surface monolayer of Caco‐2 cells. Diffusing ROS do not cause harm in adjacent (dark) regions because there the protecting character of AcYF is dominant.

## 3 Conclusion

The current paper shows that the semiconductor surfactant AcYF is willingly taken up by cells where it contributes to the reduction of oxidative stress. However, AcYF is a trojan horse as it can also produce significant amount of ROS when it becomes irradiated. Therefore, the reported surfactant class is a candidate for the photodynamic treatment, e.g., of cancer or for the protection of surfaces against the colonization by bacteria. However, yet the vesicular capsules (Scheme 1) were empty. Thus, one can envision a combination of drug‐delivery and photodynamic therapy, or using the vesicles for nanoscaled reaction vessels for photo‐driven reaction under special confinement conditions. The demonstrated possibility to excite the fluorescence as well as the ROS activity by a nonlinear two‐photon process at NIR wavelength enables 3D resolution of the excitation and higher penetration depth, which needs to be explored in 3D tissues in the future.

AcYF enters the cells but this is yet very unspecific. One can imagine to use the terminal amino group of the acridine yellow residue, or alternative functional groups in other dye molecules, to attach biologically more specific ligands. Then, one could specifically attach AcYF in particular areas of cells, and to study very locally the effects of oxidative stress.

## 4 Experimental Section

### General Procedures

Synthesis which required inert atmosphere were performed using standard Schlenk techniques under inert nitrogen atmosphere. All chemicals were purchased from commercial sources and used without further purification unless stated differently. C60 was purchased from MST (modern Synthesis Technology) with a purity of 99.9%.

### Surfactant Preparation

The synthesis was performed based on the protocols by Kunkel et al.^[^ ^14b^ ^]^

### Synthesis of Hexachlorofullerene (C_60_Cl_6_)

200 mg (0.28 mmol) of Buckminsterfullerene (C_60_) was solved in 14 mL chlorobenzene and sonicated for 10 min. 0.6 mL (6.95 mmol) Iodine monochloride were added in one shot and instantly after addition the solvent was removed under vacuum. The remaining red/brown solid was purified by column chromatography (silica gel, eluent: toluene) to yield 67% C_60_Cl_6_.

### Synthesis of ACYF

200 mg C_60_Cl_6_ and 0.485 mg Acridine yellow • HCL were solved in 40 mL water and 8 mL chlorobenzene. 0.4 mL Tetrabuthylammonium chloride (40% in water) was added together with 0.8 g NaOH and 1.5 mL H_2_O_2_ (30%) and the mixture was heated to reflux for 3 h. The mixture was allowed to cool to room temperature and 20 mL of water was added, the mixture was filtered and the precipitate was washed with water. The aqueous phase is poured into methanol and the precipitate was collected to yield the desired product as a brown solid with a yield of 58%. ATR‐IR: 3212, 2982, 2909, 2846, 1579, 1381, 1263, 1029, 960, 801. TGA: 30–195 °C 11%; 195–530 °C 17%; 530–1000; 72%.

### Analytical Methods

Attenuated total reflection‐infrared (ATR‐IR) spectra were measured with a Bruker Tensor27 spectrometer. UV–Vis Spectra were measured on an Agilent Cary 4000 spectrophotometer. Fluorescence spectra were measured using an Agilent Cary Eclipse Photospectrometer. Quantum yield measurements were carried out using a FLS 1000 from Edinburgh Instruments integrating sphere at 465 nm and calculated using the Fluoracle software from Edinburgh Instruments. All optical measurements in solution were carried out in deionized water or phosphate buffered saline (PBS) using QS high Precision Cells made of quartz glass acquired from HellmaAnalytics. Dynamic light scattering was performed on a Malvern Zen5600. Transmission electron microscopy (TEM) was performed on a Hitachi HT7800. For cryo transmission electron microscopy 2 µL of sample solution were put on a lacey carbon filmed copper grid (Plano GmbH, Muenchen). Most of the liquid was removed by blotting paper in a Leica EM GP2 (Wetzlar, Germany) grid plunge device leaving a thin water film in the water saturated atmosphere of the environmental chamber. The sample was shock frozen in liquid ethane to approximately 97 K in the liquid nitrogen cooled freezing unit of the Leica EM GP2. The sample was inserted into a cryotransfer holder (Model 698, Gatan, Muenchen) and transferred into the Transmission electron microscope.

### Nitroblue Tetrazoluim Assay

1 mL of a mixture containing PMS (nicotinamide adenine dinucleotide (NADH) (30 µm), NADH (146 µm), and NBT (100 µm) and varying concentrations of fulleren surfactant in PBS (20 mm, pH 7.4) was incubated for 5 min at room temperature and the absorbance was measured at 560 nm against the corresponding blanc fullerene surfactant sample. Each test was performed three times.

### Biological Experiments—Cell Culture

Caco‐2 cells were cultured in tissue‐coated petri dishes at 37 °C in a humidified atmosphere containing 5% CO_2_. The cells were grown in Dulbecco's MEM F‐12 (Biowest, Nuaillé, France) media supplemented with 10% foetal bovine serum (Biowest) 1 mg mL^−1^ penicillin and 0.1 mg mL^−1^ streptomycin (Merck). The culture media was renewed every 2–3 days. When a confluence of 80–90% was reached, the cells were split.

### Loading and Visualization of AcYF in the Cells

Caco‐2 (3 × 104) were seeded on a collagen‐I coated coverslips (diameter 10 mm) and cultivated for 2 days as described above. Thereafter the culture media was replaced by a fresh culture media containing 100 µm AcYF or 2 or 5 h. After addition of Hoechst (1 µm) the cells were incubated for a further hour followed by a washing with PBS at room temperature. The cover slip with the cells was transferred in a chamber filled with fresh PBS and mounted on a Nikon Eclipse TE2000‐E inverse confocal laser scanning microscope (Nikon GmbH, Düsseldorf, Germany) for observation using a 60 x water immersion objective and the software EZ‐C1 (Nikon GmbH).

All images were processed with ImageJ/Fiji. The number of plaques of AcYF in the cells was determined with the Analyze Particle function of ImageJ. For this purpose, the images were duplicated. One duplicate was used to generate a binary image. Coherent particles were identified and divided by the Watershed function of ImageJ/Fiji. Using the binary image as a reference, the particles were determined with the size: 0.34 – Infinity pixel2. The number and the area of plaques were normalized to the number of cells.

### TEER Measurement

The barrier function was assessed by measuring the TEER using the cellZscope (nanoAnalytics, Münster, Germany). The cells were seeded in transwell inserts with a 0.3 cm^2^ transparent porous (pore diameter 0.4 µm) PET membrane (BD Falcon, Corning) at a density of 3 × 10^4^ cells per insert. After cultivation period of 2 days later, the inserts with grown cells were transferred into cellZscope with fresh medium for an automatic contentious monitoring of the TEER. After a constant TEER value (600–800 Ω cm^2^) was reached after about 4–5 days, 100 µm AcYF was added apical to the culture. After an uptake time of 6 h, AcYF containing culture medium was removed and replaced by a fresh culture medium. After an equilibration time of 2 h, the cells were treated with H_2_O_2_ (Roth) diluted in cell culture medium at the appropriated concentration. For the monitoring of the TEER, the cellZscope software was used.

### AcYF‐Mediated Laser Perforation (AcYFME LP)

For the production of reactive oxygen species, confluent Caco‐2 cells cultivate in a 96‐multiwell plate were loaded for 6 h with 100 µm AcYF. After loading the plate was transferred to the laser perforator (Laser Zentrum Hannover, Hannover, Germany) and exposed to a 20 kHz pulsed laser (532 nm) with 30 mW (mJ cm^−2^) at 5 mm s^−1^ as described in Becker et al. 2018.^[^ ^23^, ^26^ ^]^ The cells were then transferred in culture incubator for 1 h to recover from the optical treatment. Thereafter Propidium iodid (10 µm) and Hoechst 33 258 (Sigma‐Aldrich) were added to the treated cells for 30 min at 37 °C. After a washing step with PBS, the cells were fixed with 4 % formaldehyde for 20 min at 4 °C followed by a washing step. Cells were stored in PBS at 4 °C for a later observation with Ti‐E inverted fluorescence microscope, the uptake of Hoechst and propidium iodide in the cells were detected with a 10 x objective and Nikon Software Nis‐Elements 4.4 (346 nm for Hoechst 33 258 and 535 nm for PI).

The uptake of propidium iodide (PI) and Hoechst 33 258 was determined with the Analyze Particle function of ImageJ/Fiji. The total cell number and the number of the destroyed cells were evaluated by counting respectively all Hoechst positive cells and Hoechst as well PI positive cells.

### Multiphoton Microscopy and NIR ROS Induction

For multiphoton microscopy (MPM) and near‐infrared (NIR) ROS induction, Caco‐2 cells were cultured in 35 mm microscopy dishes (ibidi GmbH, Germany) and incubated with 0, 25, or 100 µm AcYF, respectively, for 6 h. Some samples were additionally stained with Hoechst 33 258 for 30 min. The control group without AcYF was stained with 1 µg mL^−1^ Acridine orange for 10 min prior to imaging/treatment, to exclude unspecific phototoxic effects due to the light absorption of the dye and to allow morphological evaluation of the cells. Before imaging, the cells were washed twice with PBS to reduce the amount of free AcYF or Acridine orange, respectively, which exhibited a strong background signal. MPM was performed with a commercial two‐photon microscope (TriM Scope II, LaVision BioTec, Germany) equipped with a tunable femtosecond (fs) laser source (Chameleon Ultra II, Coherent, USA), delivering 140 fs pulses at a repetition rate of 80 MHz. The excitation wavelength was kept constant at 800 nm, which provided a good fluorescent signal in both channels. The laser was focused by a 20x objective onto the sample. Fluorescent light was collected in backward direction and split onto two photomultiplier tubes (PMTs) by a dichroitic mirror with a cut‐off wavelength of 495 nm. The PMTs were equipped with additional bandpass filters (520 ± 16 nm and 460 ± 50 nm, respectively). For each sample, a 3D stack with a step size of 1 µm was captured before treatment. Then, the samples were irradiated to induce ROS production as described below. Finally, a new stack from the same position was acquired. If not stated differently, the 3D data are presented as maximum intensity projection of the stack.

The excitation of AcYF for ROS production using near infrared (NIR) light was conducted using the same setup as for imaging. The laser wavelength for ROS production was set to 950 nm and the laser power was set to 100%, which corresponds to about 5.20 mW the microscope output. The focal area was set to match the middle height of the cells and this plane was constantly scanned for a set time span.

To ensure that the dye was excited by multiphoton absorption, a power‐signal curve was measured. Mean pixel gray values were calculated using ImageJ (https://imagej.nih.gov/ij/). The power at the microscopes exit was measured using a FieldMax II Power meter equipped with a PM10 thermophile power sensor (Coherent Inc., USA). The data were fitted with a power function to extract the photon order.

### Statistical Analysis

All data are given as mean for at least three independent experiments. The Student's paired two‐sided *t*‐test was applied to estimate the statistical significant differences between the control and the corresponding experimental conditions (*: *p* < 0.05, **: *p* < 0.01, ***: *p* < 0.001).

## Conflict of Interest

The authors declare no conflict of interest.

## Supporting information

Supporting Information

Supporting Information

Supplemental Movie 1

Supplemental Movie 2

Supplemental Movie 3

## Data Availability

The data that support the findings of this study are available in the supplementary material of this article.
