# Supplementary material for: Surfactant Semiconductors as Trojan Horses in Cell‐Membranes for On‐Demand and Spatial Regulation of Oxidative Stress
Source: Adv Healthc Mater. 2023 Jan 13;12(10):2202290. doi: 10.1002/adhm.202202290 (PMC11468338; doi:10.1002/adhm.202202290)

ADVANCED HEALTHCARE MATERIALS

Supporting Information

for *Adv. Healthcare Mater.*, DOI 10.1002/adhm.202202290

Surfactant Semiconductors as Trojan Horses in Cell-Membranes for On-Demand and Spatial Regulation of Oxidative Stress

Marian Jaschke, Masina Plenge, Marius Kunkel, Tina Lehrich, Julia Schmidt, Kilian Stöckemann, Dag Heinemann, Stephan Siroky, Anaclet Ngezahayo and Sebastian Polarz**

Supporting Information

Surfactant semiconductors as trojan horses in cell-membranes for on-demand and spatial regulation of oxidative stress

Marian Jaschke,[a,‡] Masina Plenge, [b,‡] Marius Kunkel,[c] Tina Lehrich,[b] Julia Schmidt,[b] Kilian Stöckemann,[d] Dag Heinemann,[d] Stephan Siroky,[a] Anaclet Ngezahayo,[b,] Sebastian Polarz[a,*]*

Figure S1. Amphiphilic behavior of AcYF. Concentration-dependent surface tension measurement.

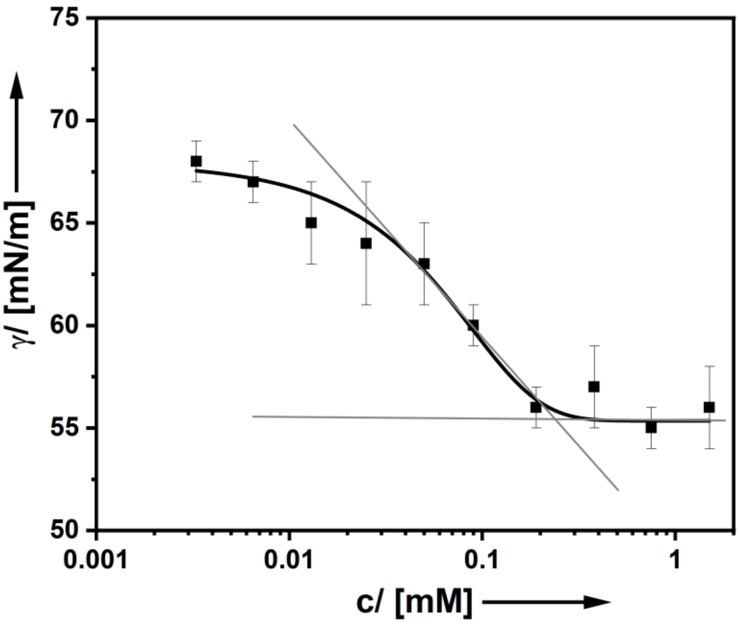

Figure S2. RNO assay to test for the photocatalytic production of singlet oxygen. t = 0 min (black), 15 min (red), 30 min (blue).

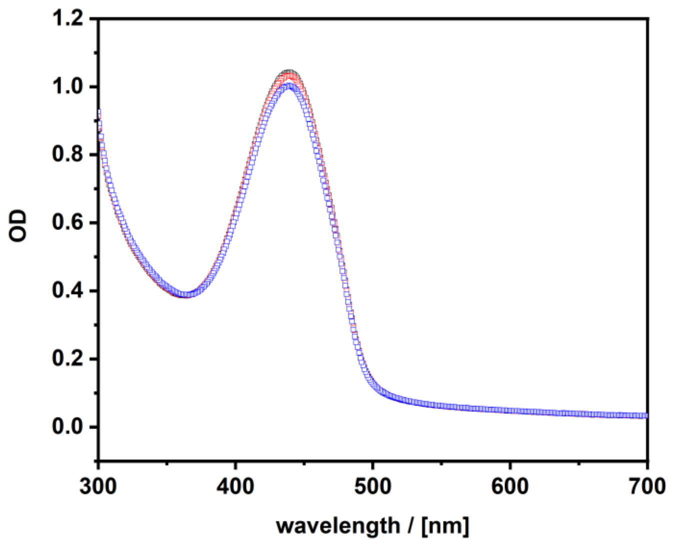

Figure S3. Stability of AcYF against photo-induced bleaching. Radiation with white LEDs (100 W); t = 0 min (black), 20 min (blue).

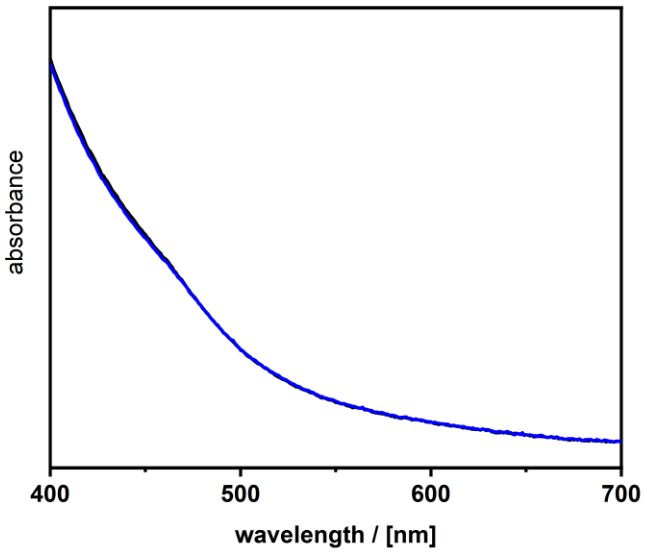

Figure S4. Non-linear excitation of AcYF surfactants. (a) Caco-2 cell containing AcYF surfactants have been imaged with a multiphoton microscope (MPM) before and after consecutive cycles of NIR laser irradiation at 950 nm (upper row). After 1 min of irradiation, first cells show formation of apoptotic vesicles (yellow arrows). After 10 min of irradiation, most cells in the irradiated area have an apoptotic morphology and multiple apoptotic vesicles are formed. In the control group, cells were stained with Acridine orange instead of AcYF, to exclude unspecific phototoxicity (lower row). Besides some motility, no signs of cellular reaction were observed. (b) Zoomed view of different z-planes from the AcYF sample after 10 min of irradiation shown in (a). The decay of the cells into multiple apoptotic vesicles is clearly visible. In the lowest plane, remaining AcFY niosomes become visible as bright spots. (c) The power/signal function yields a photon order of approx. 2, supporting the two-photon excitation of the AYF fluorescence.

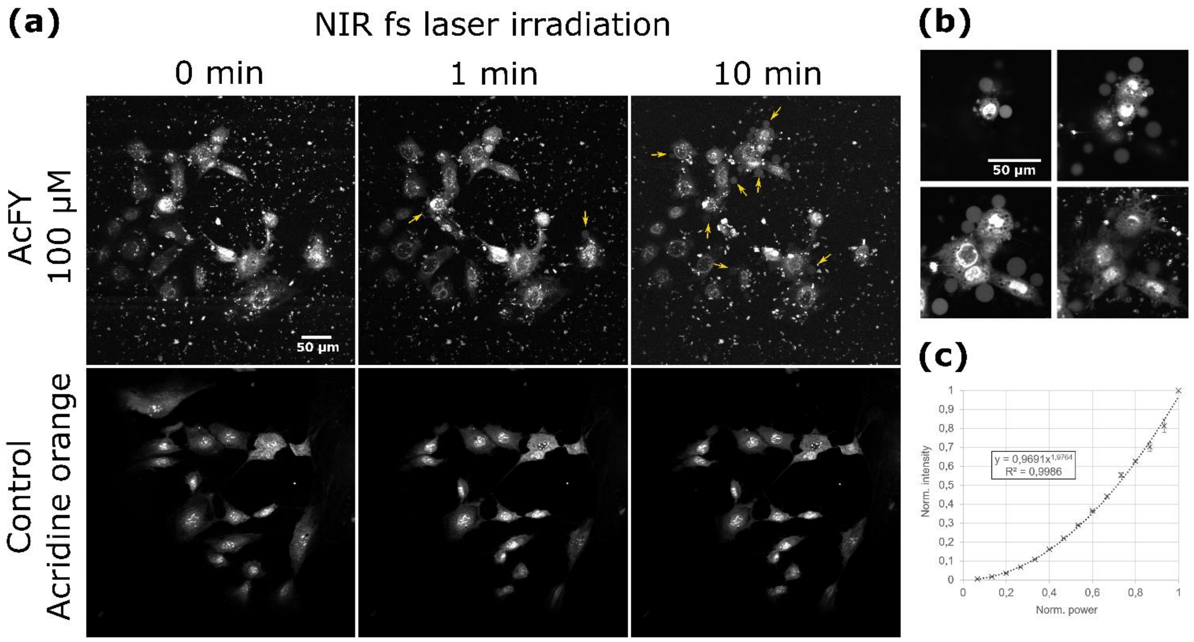

Figure S5: Long time effect of AcYF on the Caco-2 cells. The Confocal laser scanning micrographs are merged images of the transmission image and fluorescence images of Caco-2 cells cultivated in presence 100 µM AcYF for 48 h (upper row) and 96 h (lower rows). The micrographs were taken after the removal of AcYF from the culture media (left rows) and after cultivation for further 96 h (right rows) without AcYF. The blue color shows the nuclei stained by the DNA chromophore Hoechst 33258. Note the presence of mitotic cells (indicated by the red arrows), which suggests that AcYF did not affect the proliferation of the cells.

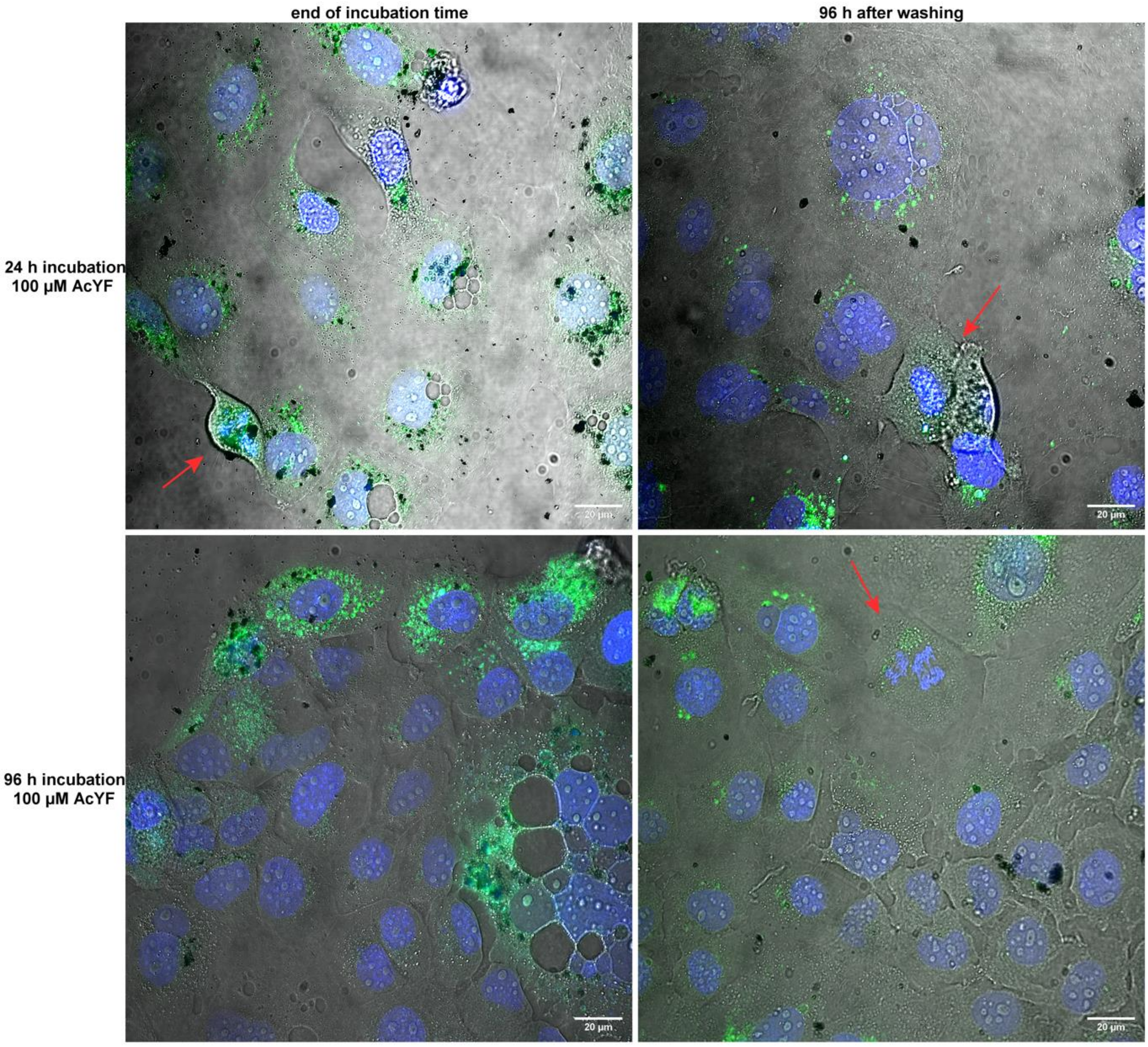

Figure S6: Fullerenol ($C_{12}F$) protects the cells from acridine yellow (AcY) related phototoxicity. The micrographs show PI-uptake (red) in cells loaded (for 6 h) with 100 µM $C_{12}F$ (upper row), 500 µm AcY (500 µM) without $C_{12}F$ (middle row) or with 100 µM $C_{12}F$ together with 500 µM AcY. The cells were treated with laser (532 nm) either in form of a scrape (middle column) or over the whole culture well (right column). Irradiation caused an increased PI-uptake in cells loaded with AcY (middle and lower row) suggesting a permeabilization of the cell membrane. Note an increased dye uptake in non-irradiated cells when AcY was present. $C_{12}F$ attenuated this effect (left, low image), so that in scrape irradiation experiments the scrape was clearly identifiable (middle, low image).

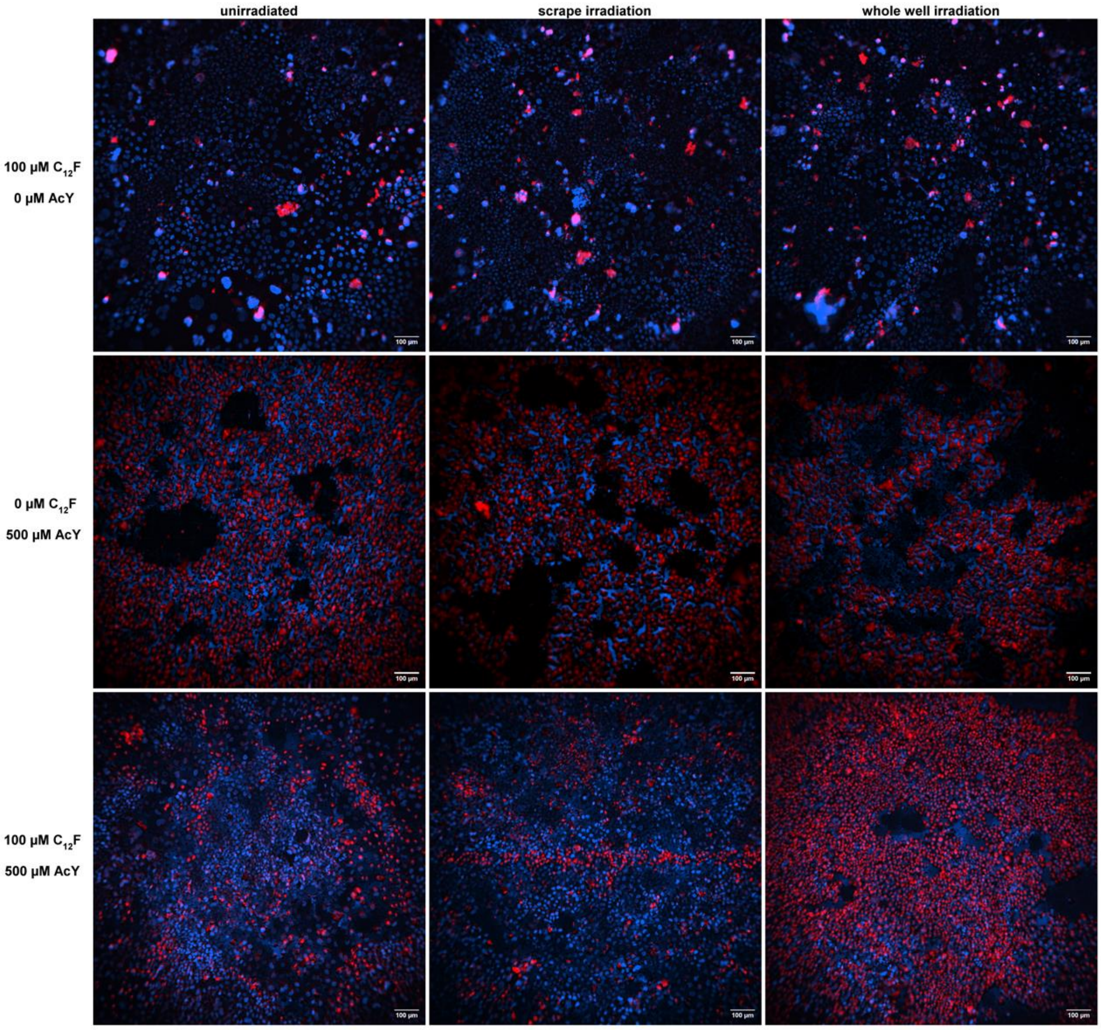

Supplement: Supplementary file 1 — Supporting Information [file ADHM-12-2202290-s005.pdf]
