# Supplementary material for: Surfactant Semiconductors as Trojan Horses in Cell‐Membranes for On‐Demand and Spatial Regulation of Oxidative Stress
Source: Adv Healthc Mater. 2023 Jan 13;12(10):2202290. doi: 10.1002/adhm.202202290 (PMC11468338; doi:10.1002/adhm.202202290)
Supplement: Supplementary file 2 — Supporting Information [file ADHM-12-2202290-s004.pdf]

ADVANCED HEALTHCARE MATERIALS

Supporting Information

for *Adv. Healthcare Mater.*, DOI 10.1002/adhm.202202290

Surfactant Semiconductors as Trojan Horses in Cell-Membranes for On-Demand and Spatial Regulation of Oxidative Stress

Marian Jaschke, Masina Plenge, Marius Kunkel, Tina Lehrich, Julia Schmidt, Kilian Stöckemann, Dag Heinemann, Stephan Siroky, Anaclet Ngezahayo and Sebastian Polarz**

Movie 1 - 20200317_Caco-2_control.mov

The movie shows a microscopy analysis of the mobility of Caco-2 cells when no surfactant is present; reference state. The images of the movies were taken on a 3D Holo-Tomographic Live Cell Imaging Microscope (Nanolive SA, Tolochenaz, Switzerland)

Movie 2 - 20210512_Caco-2_P13_C60-ACY.mov

The movie shows a microscopy analysis of the mobility of Caco-2 cells when the surfactant AcYF is present in low concentration below cac for 24h. AcYF did not affect the morphology or the motility of the cells. The images of the movies were taken on a 3D Holo-Tomographic Live Cell Imaging Microscope (Nanolive SA, Tolochenaz, Switzerland)

Movie 3 - 20210805_Caco-2_P76_ACY.mov

The movie shows a microscopy analysis of the mobility of Caco-2 cells when the surfactant AcYF is present in high concentration above cac for 24h. AcYF did not affect the morphology or the motility of the cells. Note the mitosis of an AcYF-loaded cell. The images of the movies were taken on a 3D Holo-Tomographic Live Cell Imaging Microscope (Nanolive SA, Tolochenaz, Switzerland)
